# Effects of early life exposure to traffic-related air pollution on brain development in juvenile Sprague-Dawley rats

**DOI:** 10.1038/s41398-020-0845-3

**Published:** 2020-05-27

**Authors:** Kelley T. Patten, Eduardo A. González, Anthony Valenzuela, Elizabeth Berg, Christopher Wallis, Joel R. Garbow, Jill L. Silverman, Keith J. Bein, Anthony S. Wexler, Pamela J. Lein

**Affiliations:** 1 Molecular Biosciences, UC Davis School of Veterinary Medicine, Davis, CA USA; 2 Psychiatry, UC Davis School of Medicine, Sacramento, CA USA; 3 Air Quality Research Center, UC Davis, Davis, CA USA; 4 Mallinckrodt Institute of Radiology, Washington University in St. Louis, St. Louis, MO USA; 5 The MIND Institute, UC Davis School of Medicine, Sacramento, CA USA; 6 Center for Health and the Environment, UC Davis, Davis, CA USA; 7 Mechanical and Aerospace Engineering, Civil and Environmental Engineering, and Land, Air and Water Resources, UC Davis, Davis, CA USA

**Keywords:** Molecular neuroscience, Pathogenesis

## Abstract

Epidemiological studies link traffic-related air pollution (TRAP) to increased risk for various neurodevelopmental disorders (NDDs); however, there are limited preclinical data demonstrating a causal relationship between TRAP and adverse neurodevelopmental outcomes. Moreover, much of the preclinical literature reports effects of concentrated ambient particles or diesel exhaust that do not recapitulate the complexity of real-world TRAP exposures. To assess the developmental neurotoxicity of more realistic TRAP exposures, we exposed male and female rats during gestation and early postnatal development to TRAP drawn directly from a traffic tunnel in Northern California and delivered to animals in real-time. We compared NDD-relevant neuropathological outcomes at postnatal days 51–55 in TRAP-exposed animals versus control subjects exposed to filtered air. As indicated by immunohistochemical analyses, TRAP significantly increased microglial infiltration in the CA1 hippocampus, but decreased astrogliosis in the dentate gyrus. TRAP exposure had no persistent effect on pro-inflammatory cytokine levels in the male or female brain, but did significantly elevate the anti-inflammatory cytokine IL-10 in females. In male rats, TRAP significantly increased hippocampal neurogenesis, while in females, TRAP increased granule cell layer width. TRAP had no effect on apoptosis in either sex. Magnetic resonance imaging revealed that TRAP-exposed females, but not males, also exhibited decreased lateral ventricular volume, which was correlated with increased granule cell layer width in the hippocampus in females. Collectively, these data indicate that exposure to real-world levels of TRAP during gestation and early postnatal development modulate neurodevelopment, corroborating epidemiological evidence of an association between TRAP exposure and increased risk of NDDs.

## Introduction

Neurodevelopmental disorders (NDDs) are a heterogeneous group of conditions characterized by altered brain development^1,2^. NDDs exact a significant toll on individuals, families, and society^3^. There is, therefore, considerable interest in identifying risk factors with the goal of modifying these to decrease the incidence and/or severity of NDDs. There is now credible evidence that environmental factors influence individual risk for NDDs^4,5^. Exposure to traffic-related air pollution (TRAP) and/or proximity to roadways during development represent environmental factors that have been associated with increased NDD risk^6–10^. TRAP has also been linked to NDD-relevant symptoms, such as cognitive impairment^11,12^, psychomotor deficits^13,14^, and hyperactivity^15^. While these associations have been reported for multiple cohorts in differing locations and across varying exposure levels, they have yet to be confirmed in an experimental model that reproduces the heterogeneous and dynamic nature of real-world TRAP to which humans are exposed.

The respirable fraction of near-roadway air pollution is primarily composed of vehicular emissions, which are complex mixtures of volatile organic compounds, nitrogen oxides, carbon monoxide, and ultrafine particles comprising metals, semi-volatile and nonvolatile organic compounds, and black/elemental carbon^16,17^. Exposure to selected TRAP components has been shown to cause various NDD-relevant outcomes in rodent models. Exposing mice to fine or ultrafine particulate matter (PM) during development increases neuroinflammatory cytokines and astrocyte activation^18,19^, alters microglia morphology^20^ and neurotransmitter levels^18^, and increases oligodendrogenesis^21^ and lateral ventricle size^18^. Developmental exposure of mice to diesel exhaust alters cortical volume^20^, disrupts cortical organization^22^, and impairs neurogenesis^23,24^.

Most animal studies published to date, however, have employed exposure paradigms that do not capture the complexity or spatiotemporal dynamics of real-world TRAP exposures. Since composition, dose, and timing of air pollution exposures may influence biological outcomes^25–27^, translating the relevance of much of the published animal literature to the human condition is problematic. To better represent human TRAP exposures in an animal model, we developed an exposure paradigm that preserves the gaseous and particulate components of real-world TRAP and captures daily fluctuations in pollutant levels. To accomplish this, we exposed male and female Sprague-Dawley rats from approximately gestational day (GD) 14 through postnatal day (PND) 47–51 to TRAP drawn directly from a freeway tunnel system^28,29^ and delivered unchanged to an exposure chamber in real-time. Age- and sex-matched littermate controls housed in the same facility were exposed to filtered air (FA). This paper compares neuropathologic outcomes of relevance to NDDs in TRAP vs. FA subjects. Companion papers compare effects of TRAP exposure and transportation stress on behavioral outcomes^30^ and describe the properties of the tunnel air pollutants (Bein, unpublished data).

## Methods

Detailed methods are provided in supplemental materials.

### Exposure Facility

We constructed a TRAP exposure facility adjacent to a major freeway tunnel system in the Bay Area of Northern California (Bein, unpublished data). Briefly, air drawn from above the eastbound exit of tunnel bore 1, containing both light and heavy-duty vehicle traffic, was delivered to airtight exposure chambers housed inside the facility vivarium. Supply air for separate but identical FA exposure chambers housed in the same vivarium was drawn from ambient air surrounding the facility and subjected to a series of emissions control technologies prior to being delivered to the animals. This facility was inspected and approved by the UC Davis Institutional Animal Care and Use Committee (IACUC).

### Animals

Animal procedures were conducted in strict compliance with IACUC-approved protocols, with careful regard for alleviation of pain and suffering. Animals were housed in controlled environmental conditions (20–26 °C; 12:12 light-dark cycle) with food and water provided *ad libitum*. Details of animal husbandry, pup development, and behavioral testing are described in a companion paper^30^. Briefly, pregnant Sprague-Dawley rats were transported to the exposure facility at GD 14 and randomly assigned to either FA or TRAP groups. Neither birth outcomes nor pup development were significantly different between FA- and TRAP-exposed animals. Shortly after birth, pups were randomly assigned to a cohort that underwent behavioral testing (*n* = 15 per group) or a cohort that did not (*n* = 6 per group, hereafter referred to as “untested”). Sample sizes were determined based on power analysis of previous data. Litter effects were carefully controlled in both cohorts. Pups were weaned at PND 21, and separately housed by sex for the remainder of the study. At approximately PND 50, animals were transported back to the UC Davis campus. After 18 h recovery, behaviorally tested animals were imaged by MR, while untested animals were euthanized as previously described^31^. Briefly, whole blood was collected via cardiac puncture, animals were transcardially perfused, and left and right brain hemispheres were collected for cytokine analyses and immunohistochemistry, respectively. Plasma was collected from whole blood and stored at −80 °C.

### Magnetic resonance imaging (MRI)

MRI experiments were performed on PND 53–55 behaviorally tested rats at the UC Davis Center for Molecular and Genomic Imaging (CMGI) using a Bruker Biospec 70/30 (7T) small-animal scanner (Bruker BioSpin MRI). Volumes of the whole brain and lateral ventricles were compared between experimental groups.

### Immunohistochemistry

Briefly, 10 μm thick brain sections were immunostained using the following primary antibodies: ionized binding adaptor molecule 1 (IBA1, catalog #19-19741, Wako; 1:1000), glial fibrillary acidic protein (GFAP, #3670 S, Cell Signaling; 1:1000), s100β (#ab52642, Abcam; 1:500), neuronal nuclei (NeuN, #MAB377, Millipore; 1:500), doublecortin (DCX, #MAB2253, Millipore; 1:500) and Ki67 (#ab15580, Abcam; 1:750). Fluorescent images were acquired at 20x on an ImageExpress MicroXL High-Content Analysis System (Molecular Devices). Fluorescence that is twice the background in negative control samples was considered positive staining. Overlapping tiles were stitched together to create final images for each hippocampal subregion (dentate gyrus, CA1, CA3). Images were analyzed using ImageJ v1.52p (https://imagej.nih.gov/ij/; NIH). Reactive astrogliosis and immature neurons were quantified as the percent area within each brain region immunopositive for GFAP or DCX, respectively. Microglial cell infiltration and neurogenesis were assessed as the percentage of total cells identified by DAPI staining that were immunopositive for IBA1- or Ki67/DCX, respectively. Granule cell layer width was measured as previously described^32^. All image acquisition and analyses were performed by an investigator blinded to experimental group and sex.

### Terminal deoxynucleotidyl transferase-mediated dUTP-X nick end labeling (TUNEL)

Apoptosis was quantified by TUNEL staining per the manufacturer’s instructions (kit #11684795910, Roche) with the following modifications: (1) DNase (1000 U/ml, Promega) diluted in DNase buffer supplied by the manufacturer was applied directly to slides; (2) sections were incubated in a solution of 30 nM DAPI (Thermo Fisher Scientific) in PBS for 5 min prior to mounting in Prolong Gold (Thermo Fisher Scientific).

### Quantitative polymerase chain reaction (qPCR)

Frozen hippocampal tissue was homogenized with a hand-held homogenizer (UX-44468-25, Cole-Parmer) for 15 s and total RNA was isolated from the homogenate using TRIzol reagent (Invitrogen) according to the manufacturer’s instructions. qPCR was performed as previously described^33^. Primers obtained from Integrated DNA Technologies were designed to span exon-exon junctions using Primer-BLAST. Primer sequences and efficiencies are shown in Supplemental Table 1.

### Tissue cytokine and chemokine levels

Hippocampal cytokines were quantified using a Bio-Plex Pro^TM^ 23-plex cytokine/chemokines assay (BioRad) according to the manufacturer’s instructions with the following modifications: ~300 mg of frozen hippocampal tissue was homogenized in cell lysis buffer (BioRad) supplemented with protease inhibitor cocktail (Sigma Aldrich) diluted 1:25 in water. Samples run in duplicate at 1000 µg/ml were measured using a Luminex^TM^ 100 suspension array system (Bio-Plex 200, Bio-Rad). Analyte concentrations were calculated using a standard curve derived from reference cytokines, and the geometric means were compared for each group.

### Statistical analyses

Statistical analyses were performed using GraphPad Prism statistical software, version 7.03 (GraphPad Software, La Jolla, CA). Our primary goal was to determine the effect(s) of TRAP exposure on relevant neuropathological outcomes. However, to account for the possible influence of sex on these outcomes, we performed two-way ANOVAs to determine if sex, or the interaction of sex with exposure, affected outcomes. If there was a significant main effect of sex, or an interaction between sex and exposure, then post hoc analyses were performed on males and females separately. Post hoc tests included two-sided Student’s *t* test, or Sidak’s test with correction for multiple comparisons if there was an interaction between sex and exposure. Pair-wise comparisons were performed between groups with similar variance. If two-way ANOVA determined that sex did not affect endpoints, then male and female data were collapsed by exposure, and collapsed data are represented graphically. Correlations between neurogenic markers and lateral ventricle size were analyzed by Spearman’s correlation coefficient. Data are presented as mean ± SD, unless otherwise noted. A *p*-value < 0.05 was considered significant.

## Results

**Fig. 1:**
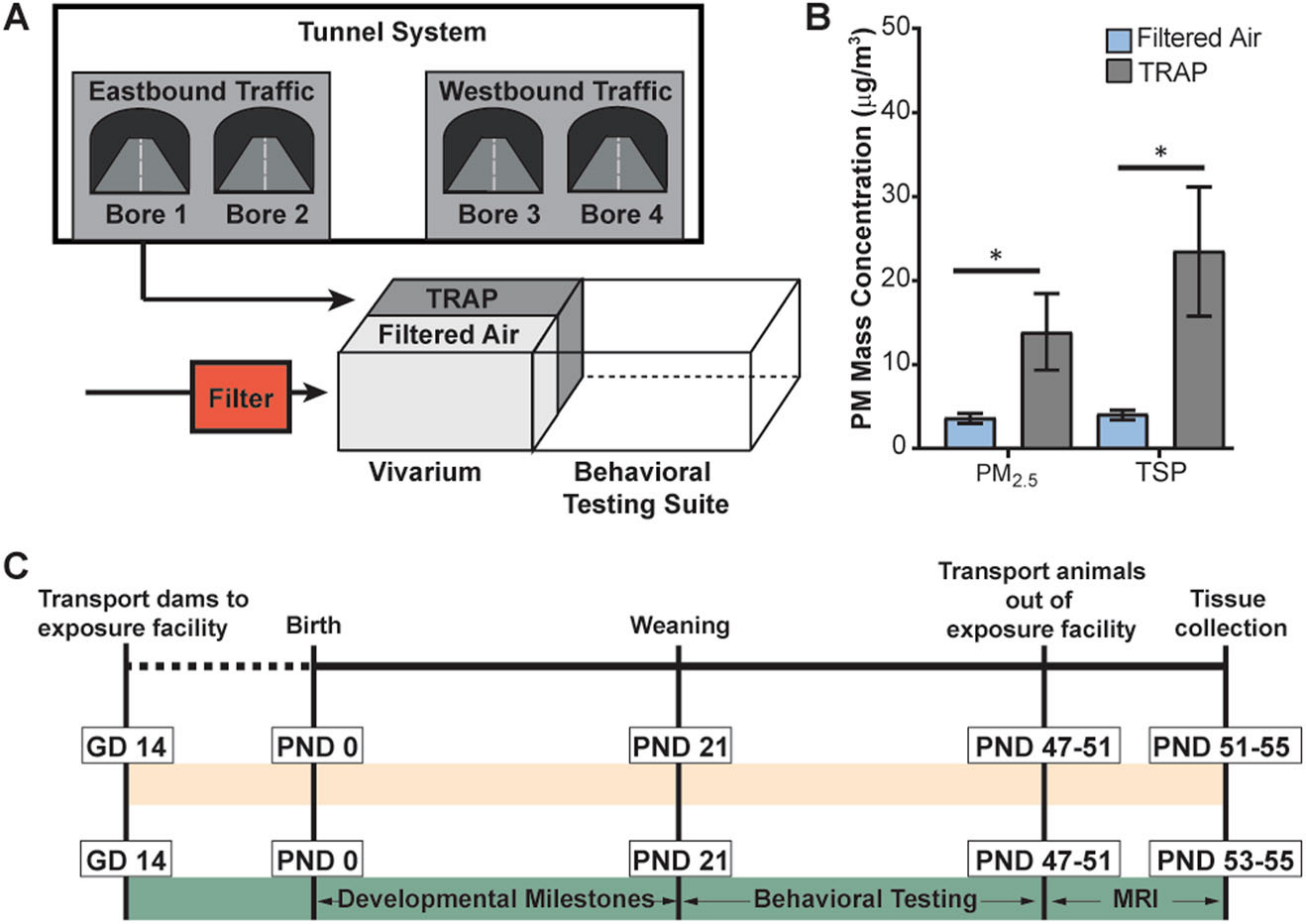
Experimental design. **a** The exposure facility consisted of a vivarium, where animals were housed in FA or TRAP exposure chambers, and a behavioral testing suite. TRAP drawn from a freeway tunnel system in Northern California was delivered unchanged to the exposure chamber. FA exposures were created by sequentially filtering ambient air adjacent to the vivarium to remove volatile and semi-volatile components, NO_x_, and ultrafine and fine particulate matter. **b** PM_2.5_ and total suspended particulates (TSP) in TRAP vs. FA shown as the mean ± SD (*n* = 8). **c** Pregnant dams were transported to the facility at gestational day 14; pups were born in the facility and remained there until PND 47–51. Pups were divided into two cohorts: one that was not used for behavioral studies (yellow bar) and another that was behaviorally tested and imaged by MR (green bar).

Male and female Sprague-Dawley rats were exposed to TRAP or FA for 24 h/d from GD 14 to PND 47–51 (Fig. 1). PM_2.5_ and total suspended particles (TSPs) were measured and averaged across 8 days within the study period. Mean PM_2.5_ levels in FA were 3.74 μg/m^3^ ± 1.21 compared to 13.94 μg/m^3^ ± 12.86 in TRAP. Mean TSP levels in FA were 4.18 μg/m^3^ ± 1.18 compared to 23.58 μg/m^3^ ± 21.48 in TRAP (Fig. 1b). More detailed descriptions of TRAP and FA PM_2.5_ composition are provided in the Supplemental Information. Briefly, elemental, organic, and total carbon levels were significantly increased in TRAP compared to FA (Supplemental Fig. 1), and traffic-related metals comprised a larger percentage of PM_2.5_ in TRAP than in FA (Supplemental Fig. 2).

### TRAP modulates the cellular neuroinflammatory response in the hippocampus

**Fig. 2:**
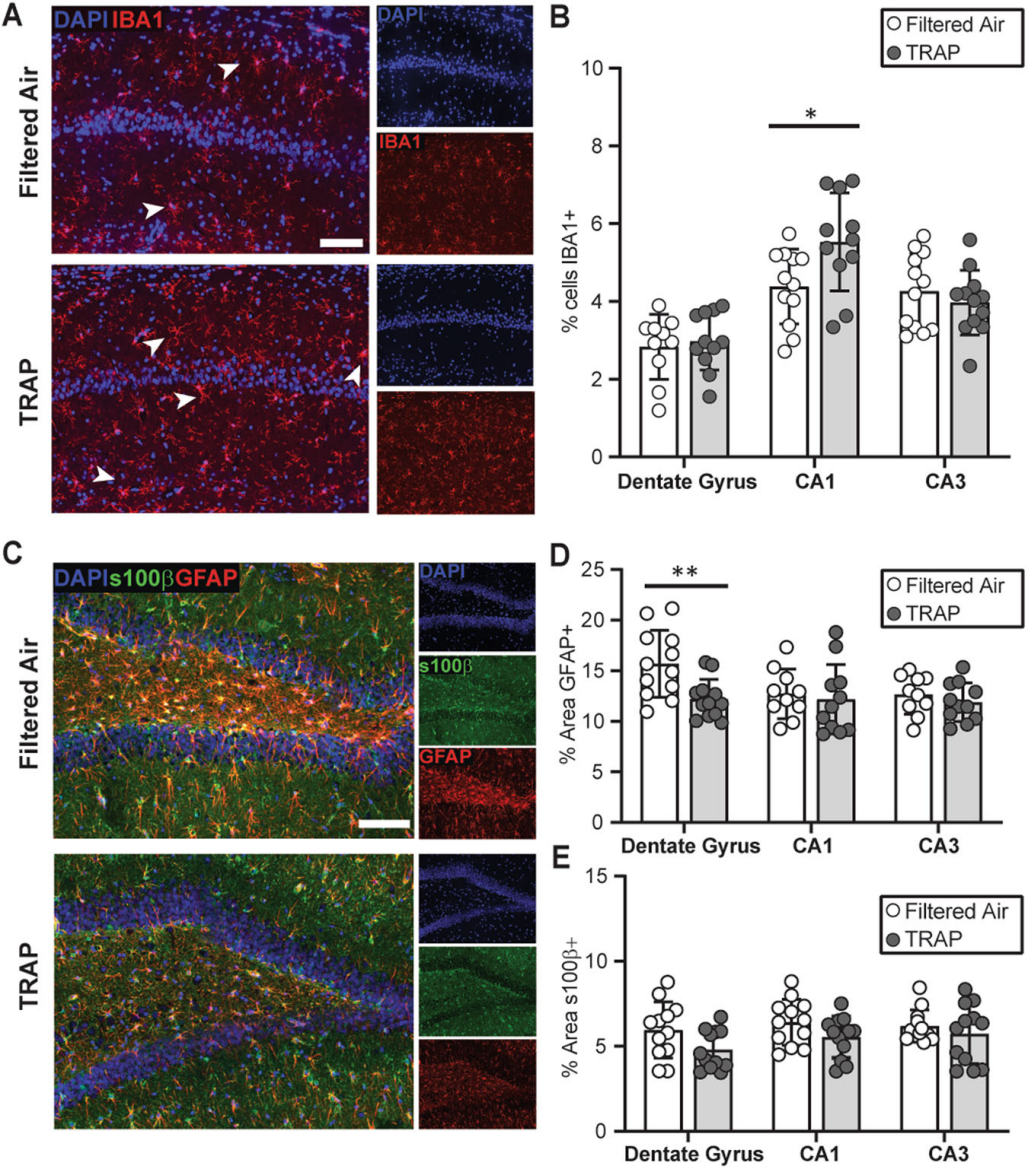
TRAP alters microglial infiltration and astrocyte reactivity at PND 51–55. **a** Representative photomicrographs of IBA1 immunoreactivity (red) in the CA1 hippocampus of FA vs. TRAP females. Sections were counterstained with DAPI (blue) to identify cell nuclei. Bar = 100 µm. Arrowheads indicate IBA1+ cells. **b** Quantification of microglial infiltration in hippocampal subregions, indicated as percentage of IBA1+ cells. **c** Representative images of GFAP (red) and s100β (green) immunoreactivity in the dentate gyrus of FA vs. TRAP females. Bar = 100 µm. Astrogliosis was quantified as the percentage area immunopositive for GFAP (**d**) or s100β (**e**). Data from male and female animals are combined and presented as the mean ± SD (*n* = 10–12 animals/exposure). Circles represent an individual animal (average of four brain slices per animal); white = FA; gray = TRAP. * = *p* < 0.05, ** = *p* < 0.01 (Student’s *t* test).

To determine whether TRAP triggers neuroinflammation, we first measured the percentage of cells immunopositive for IBA1, a biomarker of microglia^34^, in the CA1, CA3 and dentate gyrus (DG) subregions of the hippocampus in PND 51–55 rats (Fig. 2). Sex did not significantly affect the outcome in any hippocampal region (CA1: *F* (1,19) = 1.717, *p* = 0.514; DG: *F* (1,19) = 0.006, *p* = 0.938; CA3: *F* (1,20) = 0.3438, *p* = 0.564); therefore, male and female data were collapsed within exposure groups. Post hoc analysis showed that TRAP significantly increased the percentage of IBA1+ cells in the CA1 region (Fig. 2a, b) (Student’s *t* test, *p* = 0.022), suggesting TRAP induced microgliosis in this subregion. TRAP did not alter the percentage of IBA1+ cells in the DG (Fig. 2b, Student’s *t* test, *p* = 0.682) or the CA3 (Fig. 2b, Student’s *t* test, *p* = 0.427).

**Fig. 3:**
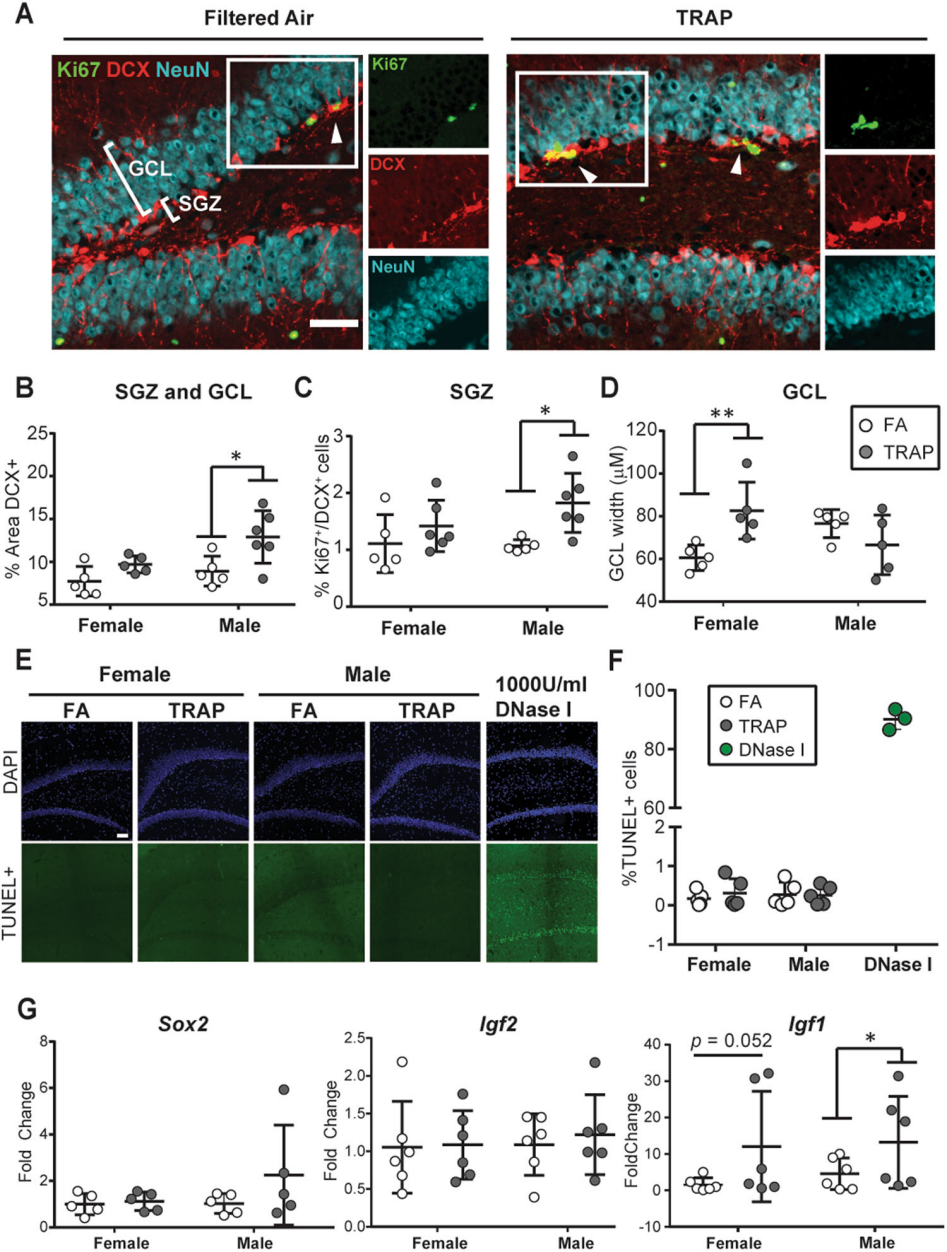
TRAP alters neurogenesis in a sex-dependent manner. **a** Representative images of the dentate gyrus from PND 51–55 males immunostained for DCX (red) to label immature neurons; Ki67 (green), proliferating cells; NeuN (cyan), mature neurons. Bar = 100 µm. Arrowheads identify cells double-labeled for Ki67/DCX; insets are of the field outlined in the white box. **b** Quantification of immature neurons measured as percent area of the SGZ and GCL immunopositive for DCX. **c** Quantification of cycling immature neurons measured as the percentage of total cells in the SGZ that were Ki67+/DCX+. **d** GCL width measured as band of NeuN immunoreactivity. **e** Representative images of TUNEL staining (green) to identify apoptotic cells; sections were counterstained for DAPI (blue) to label cell nuclei. DNase I was used as a positive control. **f** Quantification of TUNEL staining. **g** Fold-change in expression of *Sox2*, *Igf2*, and *Igf1* in the hippocampus at PND 51–55 rats, normalized to the geometric mean of *Gapdh* and *Ppia*. Data presented as mean ± SD (*n* = 5–6 animals/group). For immunohistochemistry, four sections were analyzed per animal; for qPCR, two replicates/animal. Each circle represents the mean for one animal; white = FA; gray = TRAP. *p < 0.05 (Sidak’s test for immunohistochemical data; Mann–Whitney test for qPCR data).

Astrocytes also contribute to neuroinflammatory responses^35^, therefore, we next measured immunoreactivity for the astrocyte-specific biomarkers, GFAP and S100β^36^. Analyses by 2-way ANOVA indicated that sex did not have a significant effect (*F* (1,20) = 1.557, *p* = 0.227), so male and female data were collapsed within exposure groups. We observed a significant (~22%) decrease in the area of GFAP immunoreactivity in the DG of TRAP subjects (Fig. 2c, d, Student’s *t* test, *p* = 0.0044). In contrast, TRAP had no significant effect on S100β immunoreactivity in the same brain region (Fig. 2e, Student’s *t* test, *p* = 0.058). Within the CA1 and CA3, TRAP had no significant effect on GFAP (Fig. 2d, CA1 *p* = 0.690; CA3 *p* = 0.394) or S100β (Fig. 3e, CA1 *p* = 0.110; CA3 *p* = 0.492) immunoreactivity.

### TRAP increases hippocampal IL-10 levels in females

Microglia and astrocytes influence neurodevelopment via the release of soluble signaling molecules, such as cytokines and chemokines, which may have pro- or anti-inflammatory effects, and growth factors^34^. Therefore, we next measured selected cytokine, chemokine, and growth factors in hippocampal tissue from PND 51–55 subjects using a Bio-Plex Pro^TM^ assay that quantifies protein levels of 23 analytes (Table 1). We observed sex-specific differences in the expression of several cytokines; however, only one factor, IL-10, was significantly altered by TRAP. As identified by 2-way ANOVA, there was a significant effect of sex on interleukin-6 (IL-6) levels (*F* (1,18) = 11.36, *p* = 0.003), with decreased IL-6 in males relative to females. However, TRAP did not affect IL-6 in male or females. A similar pattern was observed for growth factor granulocyte-colony stimulating factor (G-CSF): 2-way ANOVA showed a significant effect of sex (*F* (1,18) = 8.859, *p* = 0.008), with males having significantly lower levels of G-CSF than females. However, TRAP exposure did not modify G-CSF in either sex. For interleukin-10 (IL-10), we observed a significant interaction between sex and exposure (*F* (1,18) = 5.963, *p* = 0.0252). Post hoc analyses showed that TRAP females had significantly higher levels of IL-10 than FA females (Sidak’s test; *p* = 0.014), but IL-10 levels in males were not affected by TRAP (Sidak’s test; *p* = 0.417). Analysis by 2-way ANOVA also identified a significant interaction between sex and exposure for the chemokine C-C motif 2 (CCL2) (*F* (1,18) = 4.568, *p* = 0.0466); however, Sidak’s test post hoc analyses did not reveal significant effects between groups. The remaining cytokines included in the assay (Table 1) were not significantly altered by exposure or sex.

**Table 1:** Protein levels of cytokine, chemokine, and growth factors in the hippocampus at PND 51–55.

	Female (pg/ml ± s.d.)		Male (pg/ml ± s.d.)		
Analyte	Filtered Air	TRAP	Filtered Air	TRAP	Result
IL-1β	24.07 ± 5.94	30.02 ± 6.14	26.54 ± 8.40	23.33 ± 5.94	n.s.
IL-18	260.29 ± 13.81	301.76 ± 27.03	288.49 ± 87.90	260.58 ± 30.58	n.s.
IL-1ɑ	48.40 ± 8.20	51.93 ± 8.12	47.96 ± 5.85	44.82 ± 1.83	n.s.
TNFɑ	607.67 ± 123.70	665.84 ± 64.74	788.43 ± 742.93	616.39 ± 162.69	n.s.
IL-6	1396.57 ± 124.81	1496.70 ± 113.50	1329.15 ± 65.24	1280.32 ± 72.62	*p* = 0.003; Female > Male
IL-12 (p70)	541.97 ± 88.58	605.44 ± 79.65	564.53 ± 47.95	536.06 ± 40.39	n.s.
IL-17	116.80 ± 17.78	129.70 ± 12.87	120.68 ± 7.55	114.14 ± 5.02	n.s.
IL-7	196.07 ± 8.90	217.92 ± 21.67	195.50 ± 27.22	189.73 ± 32.60	n.s.
IL-2	1742.63 ± 229.40	1746.21 ± 255.39	1894.78 ± 508.37	1685.35 ± 473.38	n.s.
IFNɣ	8673.11 ± 1537.55	10204.31 ± 1875.28	9059.99 ± 1182.41	8243.57 ± 771.17	n.s.
IL-4	54.55 ± 7.35	59.55 ± 7.45	54.37 ± 6.19	53.99 ± 6.55	n.s.
IL-5	167.70 ± 17.42	180.06 ± 17.72	171.23 ± 9.62	163.38 ± 5.10	n.s.
IL-10	408.32 ± 70.37	494.47 ± 29.87	419.98 ± 46.49	382.89 ± 36.07	*p* = 0.014; TRAP Female > FA Female
IL-13	172.12 ± 26.80	190.18 ± 32.09	178.58 ± 21.29	166.68 ± 22.92	n.s.
G-CSF	22.38 ± 3.71	26.56 ± 2.87	20.77 ± 2.46	20.90 ± 2.16	*p* = 0.008; Female > Male
GM-CSF	141.74 ± 7.85	153.83 ± 14.97	138.42 ± 8.88	136.08 ± 15.87	n.s.
M-CSF	11.23 ± 2.03	12.78 ± 2.30	11.76 ± 1.65	10.61 ± 0.58	n.s.
CXCL1	55.27 ± 7.42	60.91 ± 6.42	59.48 ± 3.53	53.71 ± 2.97	n.s.
CCL3	20.36 ± 4.07	22.11 ± 3.06	21.01 ± 2.19	18.98 ± 1.05	n.s.
CCL20	9.28 ± 1.48	10.33 ± 1.30	9.31 ± 0.79	8.92 ± 0.62	n.s.
CCL5	59.28 ± 7.69	64.36 ± 6.69	62.81 ± 5.06	58.89 ± 3.47	n.s.
CCL2	153.58 ± 12.61	165.91 ± 13.02	159.85 ± 17.56	145.18 ± 16.19	*p* = 0.046; interaction sex and exposure
VEGF	316.99 ± 51.37	341.82 ± 40.88	333.32 ± 25.45	310.21 ± 20.51	n.s.

Data represent the mean ± SD (*n* = 5–6 animals/group with two technical replicates/animal). Significant main effects of sex, exposure, or interactions between sex and exposure were determined by two-way ANOVA with post hoc Sidak’s test.

### TRAP exposure increases hippocampal neurogenesis in male rats and granule cell layer width in female rats

TRAP altered GFAP, but not S100β, immunoreactivity, and this effect was observed only in the DG, which contains a neurogenic region in the postnatal rat brain^37,38^. GFAP is also a biomarker of neural progenitor cells in the rodent hippocampus^37,39^, therefore, we next considered the possibility that altered GFAP expression in the DG might reflect TRAP effects on neurogenesis. Although neurogenesis in the mammalian brain primarily occurs prior to birth, a neurogenic zone persists in the postnatal and adult subgranular zone (SGZ)^38,40^. Newborn neurons arise from neural stem cells in the SGZ, and then migrate into the granule cell layer (GCL) as they mature. To quantify neurogenesis, we first measured expression of the immature neuronal marker DCX in the SGZ and the GCL (Fig. 3a, b). Sex had a significant main effect on DCX expression (*F* (1,17) = 5.697, *p* = 0.0289), therefore, the effects of TRAP were examined separately in male and females. Post hoc analyses showed that TRAP increased DCX expression in males (Sidak’s test; *p* = 0.012), but not females (*p* = 0.291). Co-immunostaining with the cell proliferation biomarker Ki67^41^, indicated that TRAP significantly increased the percentage of Ki67^+^/DCX^+^ cells in the SGZ and GCL of males (Fig. 3a, c; Sidak’s test, *p* = 0.021). TRAP had no significant effect on the percentage of Ki67^+^/DCX^+^ in the SGZ and GCL of females (Sidak’s test, *p* = 0.451).

In order to determine whether TRAP altered expression of mature neuronal markers, we used the mature neuronal cell biomarker, NeuN^32^, to quantify GCL width at PND 51–55 (Fig. 3d). Analysis by 2-way ANOVA indicated a significant interaction between exposure and sex on GCL width (*F* (1,16) = 11.38, *p* = 0.0039). Post hoc analysis indicated that TRAP significantly increased GCL width in females (Sidak’s test; *p* = 0.009), suggesting that more mature neurons were present in the GCL of TRAP vs. FA females. There were no significant differences in GCL width between TRAP and FA males (Sidak’s test; *p* = 0.290).

To determine whether sex-specific effects of TRAP on apoptosis contributed to the sex-specific differences in TRAP effects on immature vs. mature neuronal markers, we performed TUNEL staining. Very few apoptotic cells were detected by TUNEL in the male or female DG (Fig. 3e, f). This likely reflects the developmental stage of the brain at the time of analysis, and not a technical issue with the TUNEL stain, since numerous apoptotic cells were detected in brain sections treated with DNase I as a positive control^42^. Analysis by 2-way ANOVA did not indicate any significant main effects of sex or exposure, or an interaction between these factors on apoptosis.

Neurogenesis is regulated by transcription and growth factors, including *Sox2, Igf2*, and *Igf1*^43–45^. *Sox2* is a master transcription factor that regulates neurogenesis in the DG *in utero* and during postnatal development^43^. *Sox2* transcript levels were increased in some TRAP males relative to FA males, but this effect was highly variable, and not statistically significant (Fig. 3g; Sidak’s test, *p* = 0.204). *Sox2* transcript levels were not altered by TRAP in females (Sidak’s test, *p* = 0.983). In contrast, transcript levels of *Igf1*, a growth factor implicated in autism spectrum disorder^44^ and known to enhance neurogenesis^45^, were significantly increased by TRAP in males (Fig. 3g; Mann–Whitney test, *p* = 0.039) and increased, but not significantly, in females (Fig. 3g; Mann–Whitney test, *p* = 0.052). This effect was specific to *Igf1*, as *Igf2* transcript levels were not affected by TRAP in females or males (Fig. 3g; Sidak’s test, *p* = 0.8826 and *p* = 0.9932, respectively).

### Lateral ventricle size is decreased in TRAP females

**Fig. 4:**
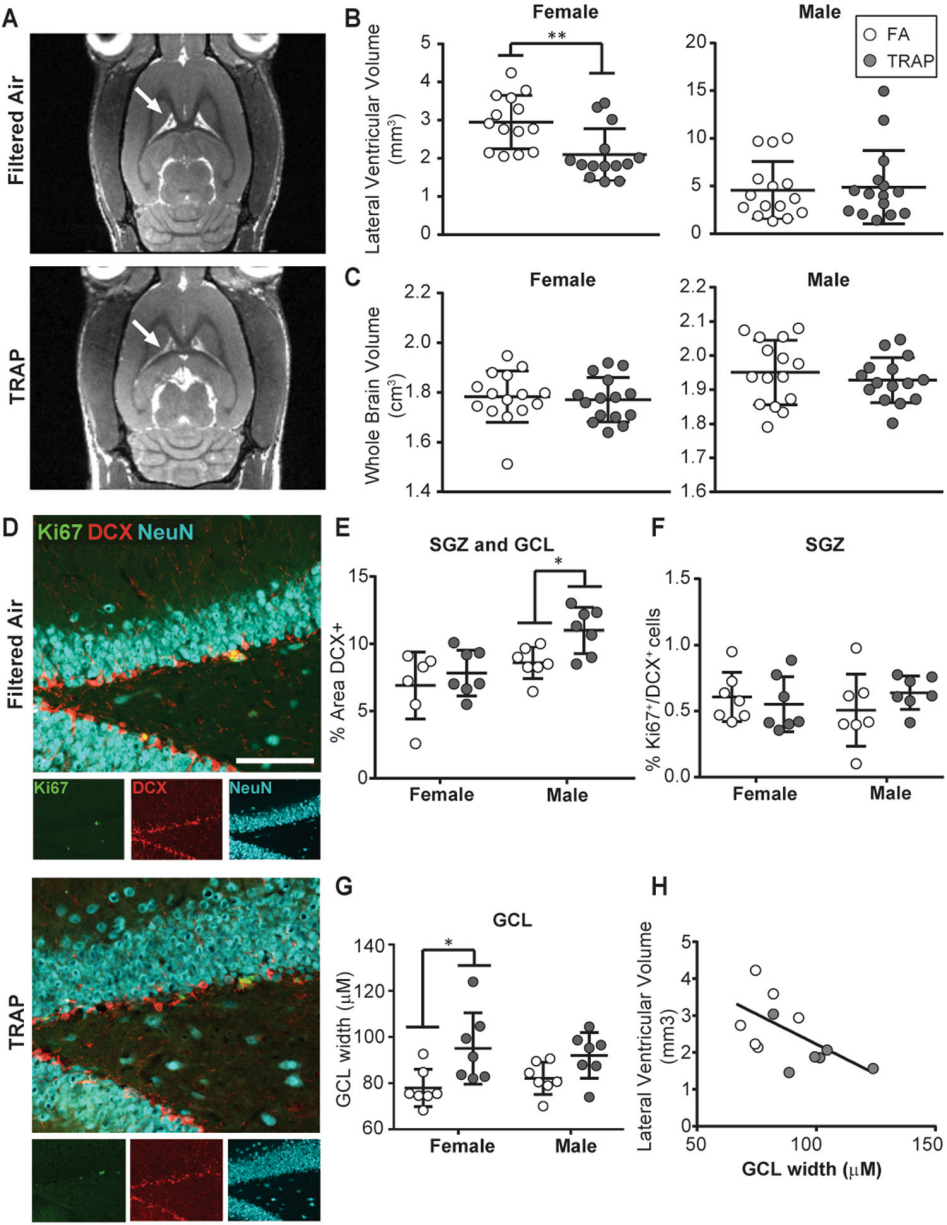
TRAP decreases lateral ventricle volume and increases GCL width in females. **a** Representative MR images of female brains at postnatal day 53-55. Arrows indicate location of lateral ventricles. **b** Lateral ventricular volumes and (**c**) whole brain volumes in TRAP vs. FA females and males. **d** Representative images of the GCL and SGZ from TRAP vs. FA females. Bar = 100 µm. **e** Quantification of immature neurons measured as percent area within the SGZ and CGL immunopositive for DCX. **f** Quantification of cycling immature neurons quantified as percentage of Ki67+/DCX+ cells. **g** Mature neuron numbers estimated as the GCL width based on NeuN immunoreactivity. **h** Correlation between lateral ventricular volume and GCL width in female rats (Spearman correlation coefficient; *r* = −0.6041, *p* = 0.0428; *n* = 11 animals). White = FA; gray = TRAP. *p < 0.05; **p < 0.01 (Student’s *t* test).

Altered lateral ventricle size has been observed in the brains of patients with autism, with studies reporting both decreases^46^ and increases^47,48^. In a mouse model, developmental exposure to ultrafine particulate matter increased lateral ventricle size^18^. To determine whether TRAP altered lateral ventricle size in our model, a cohort of subjects that previously underwent behavioral testing (Figs. 1c and 4) were imaged by MR. Analysis by 2-way ANOVA indicated that sex had a significant main effect on lateral ventricle size (*F* (1,54) = 11.22, *p* = 0.002) and brain volume (*F* (1,56) = 51.96, *p* < 0.0001). Post hoc analyses indicated that TRAP significantly decreased lateral ventricular volume in females (Fig. 4a, b; Student’s *t* test, *p* = 0.003). The decrease was not due to effects of TRAP exposure on whole-brain volume, which was not significantly different between TRAP and FA females (Fig. 4c; Student’s *t* test; *p* = 0.741). In contrast, TRAP had no significant effect on lateral ventricular volume or whole brain volume in males (Fig. 4b, c; Student’s *t* test, *p* = 0.807 and *p* = 0.708, respectively).

### Decreased lateral ventricle size is correlated with increased GCL width in TRAP females

Since behavioral testing can alter neurogenesis^49^, we tested whether the TRAP-specific changes in neurogenesis we observed in subjects that did not undergo behavior testing were also observed in the MR-imaged cohort that were tested for behavior. In the cohort of animals imaged by MR, TRAP exposure significantly increased the area of DCX immunoreactivity in males (Student’s *t* test, *p* = 0.0113), but not females (Student’s *t* test, *p* = 0.4428), indicating TRAP increased neurogenesis in males (Fig. 4d, e). However, in this cohort, TRAP did not increase the percentage of DCX^+^ cells that were also immunopositive for Ki67 in males or females (Fig. 4f; Student’s *t* test, *p* = 0.463, *p* = 0.612, respectively). GCL width was significantly increased in TRAP females vs. FA females (Fig. 4g; Student’s *t* test, *p* = 0.0236). GCL width was increased, but not significantly, in TRAP males (Student’s *t* test, *p* = 0.052).

Since decreased lateral ventricle size has been hypothesized to result from early increases in brain growth^46^, we determined whether lateral ventricle size was significantly correlated with neurogenic markers in our subjects. In females, we observed a negative relationship between lateral ventricle size and GCL width (Spearman correlation coefficient; *r* = -0.6014, *p* = 0.0428) (Fig. 4h), suggesting that the neurogenesis trends we observed by histopathology in female rats were correlated with MRI lateral ventricle volume.

## Discussion

Epidemiologic studies have linked TRAP and near-roadway exposures to increased risk of NDDs^10,11,14^, and multiple preclinical studies have linked developmental exposure to high levels of selected components of TRAP to NDD-relevant phenotypes in mice^18,20,21,50^. Here, we extend these studies to demonstrate that real-world TRAP exposures that preserve the gaseous and particulate components of TRAP, and capture the daily fluctuations in levels of these components, alter neurogenesis and growth in the developing rat brain. Specifically, we observed that TRAP: (i) increases DCX expression, a proxy measure for neurogenesis^51,52^, in the SGZ and GCL of males; (ii) increases the percentage of cycling neural progenitor cells (Ki67^+^/DCX^+^) in the SGZ of males; (iii) increases GCL width, which reflects numbers of mature neurons^32^, in females; (iv) decreases lateral ventricular volume in females; and (v) increases *Igf1* transcripts, which can promote brain growth^45,53,54^, in both males and females. These observations, which to our knowledge are the first preclinical evidence that TRAP increases neurogenesis in male rats, and increases granule cell layer width in female rats, suggest a biologically plausible mechanism by which TRAP may increase NDD risk.

Many NDDs have a strong sex bias, although the affected sex differs by disorder^55^. Epidemiologic studies have largely focused on TRAP in the context of autism spectrum disorders (ASDs) and attention-deficit hyperactivity disorder (ADHD), which are more commonly diagnosed in males^55^. Clinical studies have shown that increased brain volume in early life is characteristic of a subset of children diagnosed with ASD^56,57^, and MRI studies have shown decreased lateral ventricle size in young boys with ASD^46^, presumably as a result of brain overgrowth. In this regard, our finding of increased neurogenesis in the male brain is consistent with the human literature. While the observation of increased granule cell layer width and decreased lateral ventricle size in the female brain does not fit the sex bias profiles of ASD and ADHD, altered patterns of cellular proliferation and neuronal differentiation are common to the pathogenesis of many NDDs and intellectual disabilities that affect both males and females^1,58^. Moreover, many genetic risk factors for NDDs have functional roles in pathways that control neurogenesis^58–60^, such as *FMR1*^56^ and *PTEN*^41^. Thus, TRAP may be an environmental risk factor for NDDs in addition to ASD and ADHD.

Our observation of decreased lateral ventricle size in female but not male rats differs from several previously published studies of mice that reported increased ventricle size in both males^18^ and females^21^ in response to TRAP. These discrepant findings may reflect differences in TRAP exposure levels, windows of exposure, and species between our study and these previous studies. Alternatively, it may be that we observed sex differences in this outcome because males initially exhibit slower postnatal brain growth compared to females^57^. Thus the sex-specific effect of TRAP on brain growth phenotypes we observed at a single time point in development (PND 51–55) may reflect sex differences in intrinsic brain growth trajectories. Perhaps if we had examined later developmental times, we would have observed decreased lateral ventricles in males as well. Indeed, our MRI data indicated such a trend in TRAP males.

In contrast to our findings, two recent studies reported that TRAP decreases neurogenesis in rodent models. However, there are significant differences in the exposure paradigms between our study and these previous reports. In one study^23^, 8-week old mice were acutely exposed to diesel exhaust particles at 250–300 µg/m^3^ for 6 h, while in the second study^24^, rats were exposed to nanoparticles at 340 µg/m^3^ for 5 h/day from GD 2 to PND 175. By contrast, our peak PM_2.5_ levels varied between 18.84 and 37.68 µg/m^3^, and were delivered chronically from GD 15 to PND 50. Our window of exposure approximated mid-gestation to early adolescence in humans^57,61^, and our exposure paradigm mirrored traffic flow patterns, creating an “intermittent” exposure pattern, rather than a constant insult. Given the intermittent exposure and modest concentrations, it is perhaps not surprising that we observed TRAP to have an effect on neurogenesis that was opposite to that reported in previous studies. Cycles of injury and repair in the brain, particularly in the developing brain, have been shown to contribute to changes in circuit connectivity^62,63^, and a key determinant of circuit connectivity is neurogenesis^64,65^. It is important to note, however, that our study is consistent with one of these previous reports in that decreased neurogenesis in TRAP-exposed animals was specific to males^23^. Our finding that TRAP alters brain growth is consistent with several other studies of intermittent air pollution exposures. For example, male mice exposed to diesel exhaust particles during gestation showed increased cortical volume prior to birth, although this switched to decreased volume at PND 30^20^. Other studies have noted increased oligodendrogenesis and myelin production^21,66,67^ in male and female mice exposed to ultrafine particulate matter during gestation. Collectively, these studies underscore the importance of timing, duration, and type of exposure in dictating neurodevelopmental phenotypes.

In comparing our neuroinflammatory findings with human data, there are again similarities and differences. We observed increased IBA1+ cell density in both male and female TRAP-exposed animals, which is consistent with findings in postmortem ASD brains^68^. However, human studies report increased levels of pro-inflammatory cytokines in children diagnosed with ASD^69–71^, while the only TRAP-specific change we observed in cytokine levels was increased IL-10 in TRAP females. In the brain, IL-10 is predominantly expressed by microglia, consistent with our observation of increased microglial density in the hippocampus of TRAP animals. Since IL-10 is considered an anti-inflammatory cytokine that suppresses excessive inflammation^72^, it is possible that the IL-10 increase we observed in TRAP brains at PND 51–55 was a compensatory response to earlier inflammatory insults. Indeed, our findings echo those of other models that used intermittent exposure to ultrafine particulate matter, which reported decreased IL-1β and IL-6 in the brains of mice^18^.

The unexpected decrease in GFAP immunoreactivity that we observed in the DG of TRAP animals may reflect early perturbations in neurodevelopment. Specifically, during neurogenesis, there is an established neurogenesis-to-astrogenesis switch, in which the initial production of neurons transitions to the generation of astrocytes needed to support these neurons^36^. Although the mechanisms controlling this switch have not been fully elucidated, IL-6 signaling, neural activity, and the BMP family of growth factors are implicated^73^. It is hypothesized that interference with this process during development may result in delayed astrogenesis that contributes to the pathogenesis of NDDs^36^.

Living in close proximity to roadways increases exposure to not only TRAP, but also noise and vibration, which are both associated with maternal stress, adverse neurodevelopmental outcomes^8^, and poor cognition^74^. Since TRAP and FA animals were similarly exposed to noise and vibration in the exposure facility, it seems likely that differential outcomes between these groups were predominantly due to TRAP. However, we cannot rule out potential interactions between TRAP and noise/vibration. Another limitation of our study is that we only examined neuropathology at one time point. Thus, we cannot determine whether gestational or postnatal exposures were more important in driving TRAP-associated neurogenesis phenotypes. In humans and in rodents, the majority of neurons within the brain are generated prenatally^36,38^. After birth, neurogenesis declines rapidly, and in the adult brain, it is largely absent except in the SGZ and SVZ, where neurogenesis occurs throughout life, albeit at declining levels^38,75^. Since the majority of granule cells in the rat DG are generated in the first three postnatal weeks^76,77^, it seems plausible that postnatal exposures to TRAP are critical. However, we cannot rule out the possibility that exposure during gestation was ultimately responsible for the neurogenesis phenotypes we observed.

In conclusion, our data are among the first to link TRAP to increased neurogenesis in male rats, and increased granule cell layer width in female rats. Given other laboratory and clinical data identifying altered patterns of neurogenesis and brain growth as a pathogenic mechanism of NDDs^60,78^, our data support a model in which TRAP exposure during critical developmental windows can bias brain growth towards NDD phenotypes.

## Supplementary information

Patten et al., Supplemental Information

## Data Availability

Data from this study are available from the corresponding author on reasonable request. Supplemental information including detailed methods, supplemental figures, and supplemental tables is available from the Translational Psychiatry website.
