# Supplementary material for: Effects of early life exposure to traffic-related air pollution on brain development in juvenile Sprague-Dawley rats
Source: Transl Psychiatry. 2020 May 27;10:166. doi: 10.1038/s41398-020-0845-3 (PMC7264203; doi:10.1038/s41398-020-0845-3)
Supplement: Supplementary file 1 — Patten et al., Supplemental Information [file 41398_2020_845_MOESM1_ESM.docx]

**Supplemental Information**

(13 pages, 2 figures, 1 table)

***Effects of Early Life Exposure to Traffic-Related Air Pollution***

***on Brain Development in Juvenile Sprague-Dawley Rats***

Kelley T. Patten^1^ MA, Eduardo A. González^1^ BS, Anthony Valenzuela^1^ MS, Elizabeth Berg^2^ BS, Christopher Wallis^3^ MS, Joel R. Garbow^4^ PhD, Jill L. Silverman^2^ PhD, Keith J. Bein^3,5^ PhD, Anthony S. Wexler^3,6^ PhD, and Pamela J. Lein^1^ PhD

*^1^Molecular Biosciences, UC Davis School of Veterinary Medicine, Davis, CA, U.S.A.*

*^2^Psychiatry, UC Davis School of Medicine, Sacramento, CA, U.S.A.*

*^3^Air Quality Research Center, UC Davis, Davis, CA, U.S.A.*

*^4^Mallinckrodt Institute of Radiology, Washington University in St. Louis, St. Louis MO, U.S.A.*

*^5^Center for Health and the Environment, UC Davis, Davis, CA, U.S.A.*

*^6^Mechanical and Aerospace Engineering, Civil and Environmental Engineering, and Land, Air and Water Resources, UC Davis College of Engineering, Davis, CA, U.S.A.*

**Supplementary Methods**

*Characterization of the Exposure Atmosphere*

Physical and chemical characterization of the exposure atmospheres inside the TRAP and FA exposure chambers was accomplished via a combination of real-time air quality instrumentation and offline analysis of filter-based samples using traditional analytical techniques. Size distribution of particle number concentration was measured at 3-min resolution using a Scanning Mobility Particle Sizer (TSI, Model 3936). PM_1_, PM_2.5_, PM_10_ and TSP mass concentrations were obtained at 5-sec resolution using a DustTrak DRX Aerosol Monitor (TSI, Model 8533). Total volatile organic compounds, carbon monoxide, carbon dioxide, and nitrogen dioxide were measured at 6-sec resolution via a photoionization detector with integrated electrochemical sensors (GrayWolf, Model IQ-610). Filter-based PM samples were collected for offline gravimetric and chemical characterization using IMPROVE (Interagency Monitoring of Protected Visual Environments) PM sampler systems composed of four different sampling modules: (**A**) PM_2.5_ mass concentration via gravimetric analysis and elemental analysis via X-ray fluorescence; (**B**) PM_2.5_ molecular organic compounds via gas chromatography-mass spectrometry; (**C**) PM_2.5_ elemental and organic carbon (EC/OC) via thermal optical reflectance; and (**D**) total suspended particulates (TSP) mass concentration. Each exposure chamber has its own dedicated sampler system and modules **A**, **C** and **D** collected 24-h samples once every three days while module **B** collected contiguous 72-h samples to increase the molecular resolution of the non-targeted organics analysis. Detailed information on the design and operation of the IMPROVE sampler systems, as well as subsequent analysis of filter samples, can be found in the standard operating procedures of the IMPROVE Particle Monitoring Network (<http://vista.cira.colostate.edu/Improve/particulate-monitoring-network/>).

*Animals*

All animal procedures were conducted in strict compliance with protocols approved by the UC Davis IACUC with careful regard for alleviation of pain and suffering. Animal facilities were maintained under controlled environmental conditions (20-26 °C, 12:12 light dark cycle) with food and water provided *ad libitum*.

Two cohorts of Sprague-Dawley rats were used in this study: one that underwent behavioral testing (n=15 per group), and one that did not (n=6 per group, referred to as “untested”). Litter effects were carefully controlled in both cohorts. Animal husbandry is described in detail in a companion paper (Berg et al., submitted). Briefly, Sprague-Dawley breeders (PND 80 – 90) were obtained from Envigo (Livermore, CA) and paired for two weeks. Pregnant dams were transported to the exposure facility at approximately gestational day (GD) 14 and individually housed in standard plastic-shoebox cages, with paper bedding. Dams were indistinguishable at GD14, and were randomly assigned to either FA or TRAP exposure chambers upon arrival at the exposure facility, where they remained for the duration of the study. There were no significant differences between FA- and TRAP-exposed animals with regard to birth outcomes or the development of male and female pups, as described in a separate companion paper characterizing the behavioral development of these pups (Berg et al., submitted). At postnatal day (PND) 2, pups were marked for identification using non-toxic animal tattoo ink (Ketchum Manufacturing Inc., Brockville, ON, Canada) and a 23-gauge hypodermic needle tip. Tattoos were coded to allow researchers to perform all experiments blinded to treatment group. Pups were weaned at PND 21, and separated by sex for the remainder of the study. Behavioral testing results and methods are described in detail in a companion paper (Berg et al, 2019, submitted). Animals in the untested cohort remained in the exposure chambers throughout the study. At ~PND 50, all animals were transported back to the UC Davis campus. The age range of pups at return was PND 47 – 51, with mean and standard deviation of PND 49.54 and 1.49 days, respectively.

After an 18-h recovery from transport, animals used in behavioral assays underwent magnetic resonance imaging (described in detail below) before being euthanized. Animals in the untested cohort were immediately euthanized following anesthesia with 4% isoflurane (Southmedic Inc., Barrie ON) in a mixture of 2/3 medical grade air and 1/3 medical grade oxygen administered via inhalation at a rate of 1.5 liters/min. Blood was collected from the heart via cardiac puncture using K2EDTA-coated tubes (BD Biosciences, San Jose, CA) and tubes were inverted 10 times before being centrifuged at 1800 x g for 20 min. Plasma was transferred by plastic micropipette to individual micro centrifuge tubes and frozen at -80°C. Animals were transcardially perfused with 100 ml of cold 0.1 M phosphate buffered saline (PBS; pH 7.2, 137 mM NaCl, 10 mM sodium phosphate dibasic, 1.8 mM Potassium phosphate monobasic) at a rate of 15 ml/min using a Masterflex peristaltic pump (Cole Parmer, Vernon Hills, IL). The brain was rapidly removed and sagittally bisected in a stainless steel rat brain matrix (Zivic Instruments, Pittsburgh, PA). The left hemisphere hippocampus and cortex were removed, snap-frozen in individual tubes, and stored at -80°C. The right brain hemisphere was cut into 2 mm coronal blocks and fixed in 4% w/v paraformaldehyde (Sigma Chemical, St. Louis, MO) in PBS for 24 h at 4°C.

*Magnetic Resonance Imaging (MRI)*

MRI experiments were performed for behaviorally tested animals between PND 53-55 at the Center for Molecular and Genomic Imaging (CMGI, UC Davis) using a Bruker Biospec 70/30 (7T) small-animal scanner (Bruker BioSpin MRI; Ettlingen, Germany). This scanner includes a 116-mm internal diameter BGA-12S gradient (450 mT/m, 4500 T/m/s), a 72-mm internal diameter linear transmit coil, and a four-channel rat-brain phased array in cross-coil configuration for signal reception. Animals were maintained under isoflurane anesthesia (1-3%) in O_2_ (1-2%) during scanning on a thermoregulated stereotactic restraint (37 °C) for a maximum of 2 h. Temperature and respiration rate were monitored throughout scanning using SAII 1025 small-animal monitoring equipment (Small Animal Instruments, Inc.; Stony Brook, NY). To enhance contrast between tissue and fluid and, thereby, allow better visualization of the lateral ventricles of the brain, a 3-dimensional balanced steady-state free precession (bSSFP) sequence was used, which provides a mix of T1 and T2 weighting (1, 2). A total of eight MR scans were performed at the following phase angles to minimize image distortion (3): 45°, 90°, 135°, 180°, 225°, 270°, 315°, and 360°. The field of view for each scan was sufficient to cover the entire brain (34.95 x 22.05 x 16.05 mm). Additional MR scan parameters were as follows: TR=6.2, TE=3.1, resolution=0.15 mm isotropic, flip angle=65°. The eight brain scans were reconstructed using Paravision software v 5.1 (Bruker BioSpin MRI) and averaged using ImageJ (<https://imagej.nih.gov/ij/>; National Institutes of Health) to obtain final brain images. MR images were subsequently processed and analyzed using PMOD v 3.9 (PMOD Technologies; Zurich, Switzerland). An automated brain mask was applied to each scan using the probability brain mask tool available in PMOD. The brain mask was used to create a region of interest (ROI) and extract whole brain volume for each animal. ROIs for the lateral ventricles of the brain were created using an automated threshold tool in PMOD to delineate the ventricles based on pixel intensity. Total volumes of the whole brain and lateral ventricles (cm^3^) were extracted and compared between experimental groups.

*Immunohistochemistry*

Following fixation, coronal brain blocks were washed with PBS and placed in 30% w/v sucrose (Thermo Fisher Scientific, Waltham, MA) in PBS at 4°C until blocks sank. Blocks were then embedded in Optimal Cutting Temperature compound (OCT, Thermo Fisher Scientific) and flash frozen in a bath of dry-ice and methanol. Cryosections (10 µm thick) were cut on a Microm^TM^ HM550 cryostat (Thermo Fisher Scientific), mounted on positively charged adhesion slides (Fisher Scientific), and stored at -80°C until further processing. For immunohistochemical analyses, 3 or 4 sections from each animal’s dorsal hippocampus [Bregma -3.3 to -4.2; confirmed using a photographic atlas of the rat brain (4)] were immunostained and imaged as described below.

Brain sections were immunostained using the following primary antibodies: IBA1 (ionized binding adapter molecular 1, catalog #19-19741, 1:1000; Wako, Richmond, VA), GFAP (glial fibrillary acidic protein, catalog #3670S, 1:1000; Cell Signaling, Danvers, MA), s100β (catalog #ab52642, 1:500; Abcam, Burlingame, CA), NeuN (neuronal nuclei, catalog #MAB377, 1:500; Millipore, Billerica, MA), doublecortin (DCX, catalog #MAB2253, 1:500; Millipore) and Ki67 (catalog #ab15580, 1:750; Abcam). Antigen retrieval was performed by heating samples in 10 mM aqueous sodium citrate buffer (pH 6.0) for 30 min in a vegetable steamer (HS800, Black & Decker, Towson, MD). Sections were blocked in PBS supplemented with 10% v/v normal goat serum (Cell Signaling), 1% w/v bovine serum albumin (Thermo Fisher Scientific) and 0.03% v/v Triton X-100 (Thermo Fisher Scientific). Sections were incubated overnight at 4°C with primary antibodies diluted in blocking buffer. Following three 10 min washes in PBS with 0.03% v/v Triton X-100, sections were incubated with Alexa-Fluor® secondary antibodies (1:1000, Millipore) in PBS with 0.03% v/v Triton X-100 for 1.5 h at room temperature and mounted in Prolong Gold with DAPI (Thermo Fisher Scientific). Negative controls were treated the same as other samples, with the exception that blocking buffer was used in place of primary antibody.

Fluorescent images were acquired using a 20x objective on an ImageExpress MicroXL High-Content Analysis System (Molecular Devices, Sunnyvale, CA). Fluorescence intensity that was twice the background fluorescence in negative control samples was considered positive for each biomarker. Multiple overlapping tiles were stitched together to create final images for each brain region (dentate gyrus, CA1, CA3). All images were analyzed using ImageJ v 1.52p (<https://imagej.nih.gov/ij/>; National Institutes of Health), and data were averaged for each animal across all imaged sections. GFAP and DCX immunoreactivity were quantified as the percent immunopositive area within each brain region of interest. Microglial cell infiltration and neurogenesis were assessed as the percentage of total cells identified by DAPI staining that were immunopositive for IBA1 or Ki67/DCX, respectively. Granule cell layer width was measured as previously described (5). All image acquisition and analysis was completed by an investigator blinded to experimental group and sex. Animals were excluded from immunohistochemical analyses based on pre-established criteria (if Bregma -3.3 to -4.2 was not available), and outliers were excluded based on Grubbs' test.

*Terminal deoxynucleotidyl transferase-mediated dUTP-X nick end labeling (TUNEL)*

TUNEL staining was performed to assess apoptosis according to the manufacturer’s instructions (kit #11684795910, Roche, Mannheim, Germany) with the following modifications: (1) DNase at 1000 U/ml (Promega, Madison WI) was diluted in DNase buffer supplied by manufacturer, and applied directly to slides; (2) Slides were washed in a solution of 30 nM DAPI (Thermo Fisher Scientific) in PBS for 5 min prior to mounting in Prolong Gold (Thermo Fisher Scientific).

*Quantitative Polymerase Chain Reaction* (qPCR)

RNA isolation and qPCR was performed as previously described (6) with the following modifications: frozen hippocampal tissue was homogenized with a hand held homogenizer for 15 seconds (UX-44468-25, Cole-Parmer, Vernon Hills, IL), and total RNA was isolated using TRIzol reagent (Invitrogen, Carlsbad, CA) according to the manufacturer’s instructions. Primers that span exon-exon junctions were designed using Primer-BLAST and were obtained from Integrated DNA Technologies (IDT, San Jose, CA). Gene specific primers for the following genes are detailed with primer efficiencies in supplemental Table S1: SRY-box 2 (*Sox2)*, insulin-like growth factor 1 (*Igf1*), insulin-like growth factor 2 (*Igf2),* peptidylprolyl isomerase a (*Ppia*), hypoxanthine guanine phosphoribosyl transferase (*Hprt*). Samples were assayed in triplicate and run at the Real-Time PCR Research and Diagnostics Core Facility at UC Davis on a 7900HT Fast Real-Time PCR System (Applied Biosystems Waltham, MA). Relative mRNA expression was determined using the ΔΔCT method as described previously (7), and fold change was normalized to the geometric mean of reference genes *Ppia* and *Hprt* (7).

*Tissue Cytokine, Chemokine, and Growth-Factor Levels*

Hippocampal cytokines were quantified using a Bio-Plex Pro^TM^ 23-plex cytokine assay (BioRad, Hercules, CA) according to manufacturer’s instructions with the following modifications: ~300 mg of frozen hippocampal tissue was homogenized using a hand-held ultrasonicator (VirSonic 100, VirTis, Los Angeles, CA). Samples were homogenized in cell lysis buffer (BioRad, Hercules, CA) with protease inhibitor cocktail (Sigma Aldrich, St. Louis, MO) diluted 1:25 in water before being centrifuged at 4500 rcf for 4 min. Total protein levels were quantified using a bicinchoninic acid colorimetric assay, according to the manufacturer’s instructions (Pierce Biotechnology, Rockford IL). Samples were diluted to 1000 µg/ml in cell lysis buffer and were centrifuged at 13,000 rcf for 5 min prior to analysis. Samples were run in duplicate and were measured using a Luminex^TM^ 100 suspension array system (Bio-Plex 200, Bio-Rad, Hercules CA) at the UC Davis Intellectual and Developmental Disabilities Research Center (IDDRC) core facility. Cytokine/chemokine concentrations were calculated using a standard curve derived from reference cytokines, and the geometric means were compared for each group.

**Supplemental Table S1.** Primer designs and efficiencies for relative mRNA abundance.

|  | Forward Primer 5´→ 3´ | Reverse Primer 3´→ 5´ | Primer Efficiency |
| --- | --- | --- | --- |
| *Ppia* | TCTCTCCGTAGATGGACCTG- | ATCACGGCCGATGACGAGCC | 105.67% |
| *Hprt* | GGAGAGCGTTGGGCTTACCT | CGGCAAAAAGCGGTCTGAGG | 97.76% |
| *Sox2* | AGAACTAGACTCCGGGCGAT | ACCCAGCAAGAACCCTTTCC | 101.25% |
| *Igf1* | ATGTTCCCCCAGCTGTTTCC | TTTCGATTGCGCAGGCTCTA | 98.40% |
| *Igf2* | TGTGAGAACCTTCCAGCCTTTT | CACAAGGCGAAGGCCAAAGA | 102.12% |


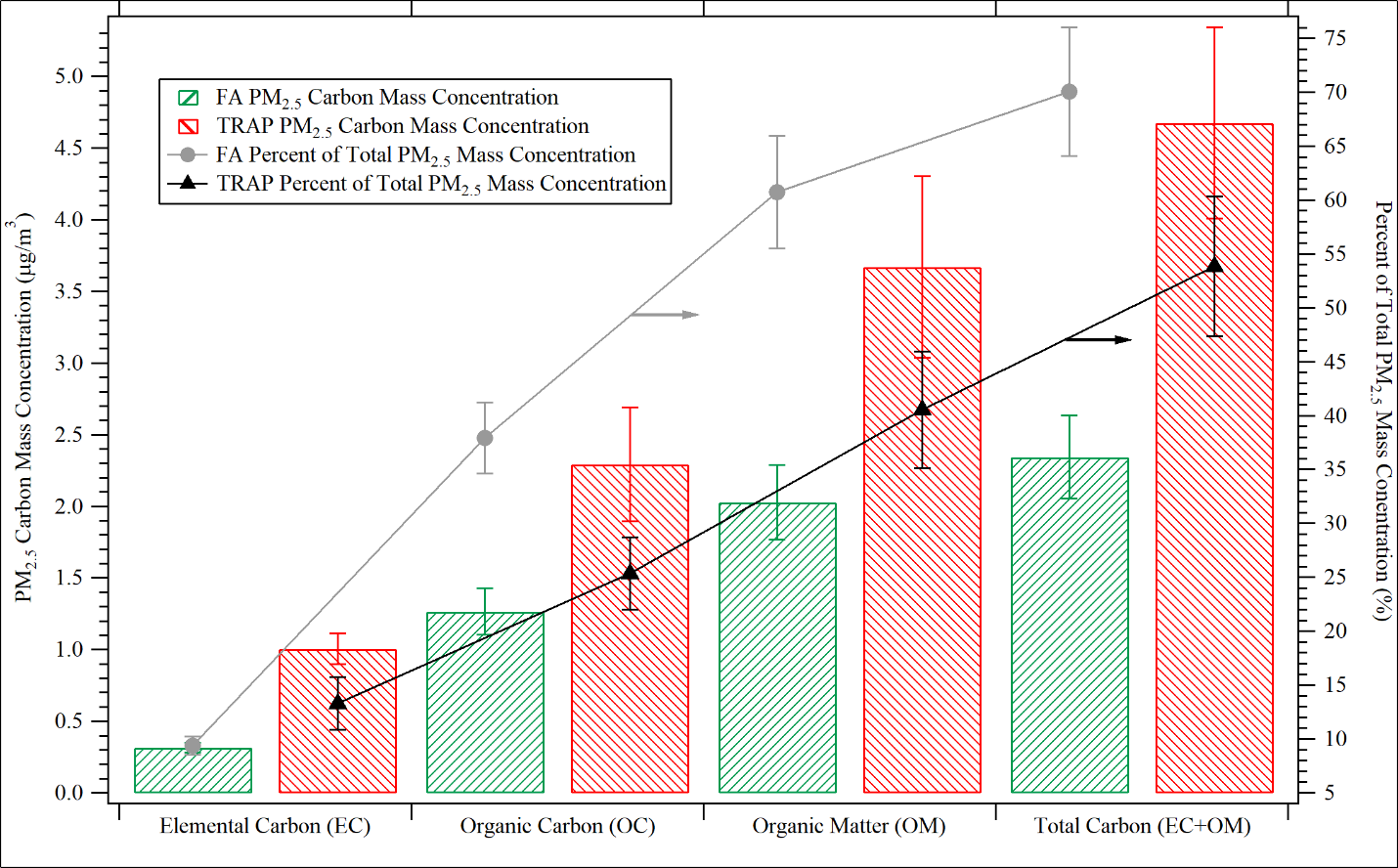


**Supplemental Figure S1**. *Bars/left axis*: study-averaged PM_2.5_ mass concentrations of Elemental Carbon (EC), Organic Carbon (OC), Organic Matter (OM) and Total Carbon (EC+OM) determined via Thermal Optical Reflectance (TOR) analysis of PM samples collected immediately upstream of the Filtered Air (FA) and Traffic-Related Air Pollution (TRAP) exposure chambers at the exposure facility. Error bars represent ± standard error of the mean. *Lines/markers/right axis*: study-averaged percent of total PM_2.5_ mass concentration accounted for by each of the carbon species for the FA (gray line) and TRAP (black line) exposure chambers; error bars represent ± propagation of uncertainty in the standard error of the mean. Filter-based PM samplers (8) owned and maintained by the Interagency Monitoring of Protected Visual Environments (IMPROVE) were deployed in this study to collect 24-hour continuous PM samples every third day for the study duration. Gravimetric analysis of the collected filter samples was performed according to the standard operating procedures of the IMPROVE Particle Monitoring Network (9) and a subset of these filters were selected for PM EC and OC mass concentration measurements via TOR according to the handling, storage, measurement and QA/QC protocols of the Desert Research Institute (10). An organic molecular weight per carbon weight estimate of 1.6 ± 0.2 (11) was used to convert the OC measurements to OM estimates; total carbon is determined from the sum of EC and OM.


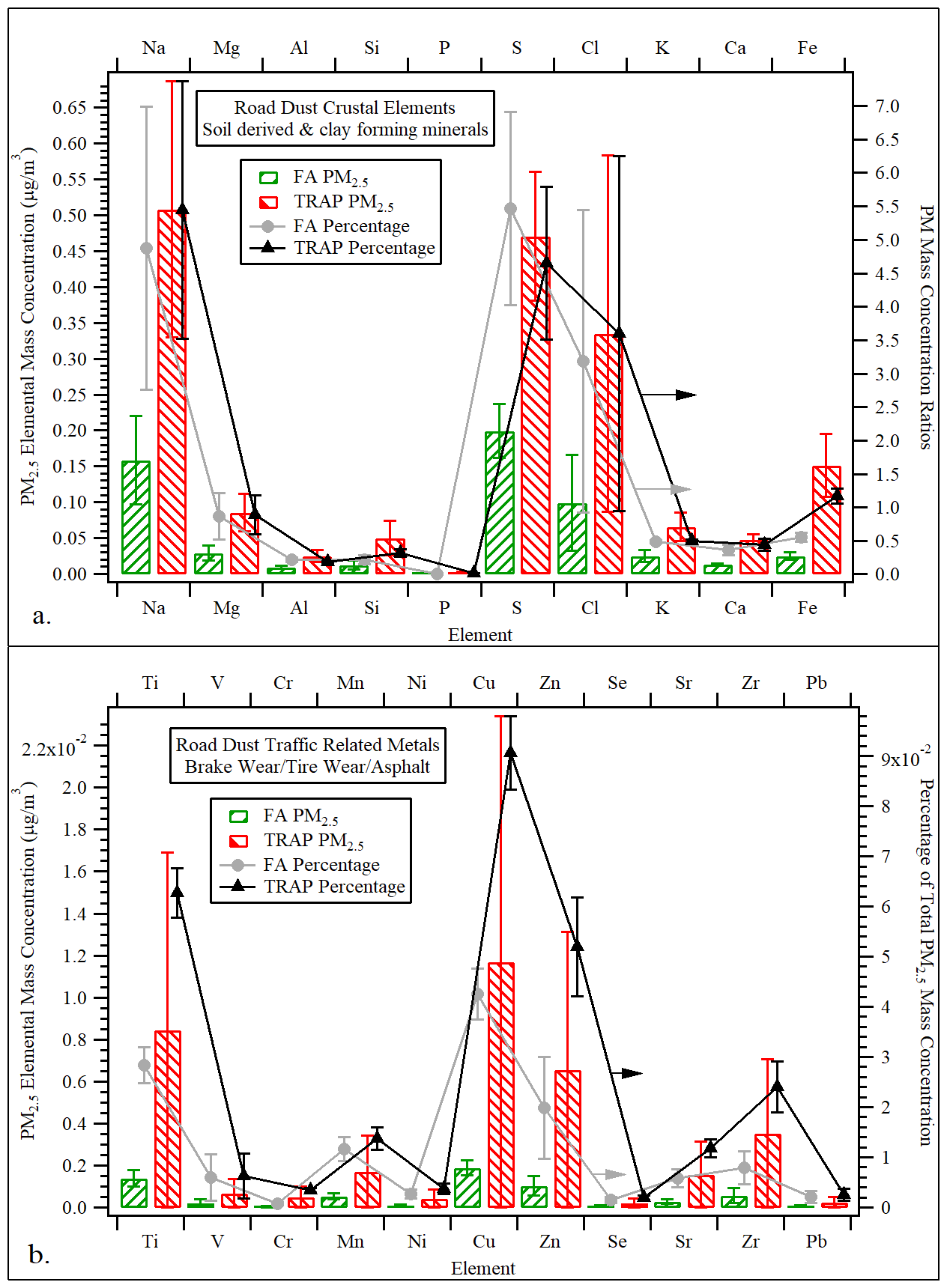


**Supplemental Figure S2.**  *Bars/left axis*: study-averaged 24-hour PM_2.5_ elemental mass concentrations for (**a**) road dust crustal elements and (**b**) traffic-related metals determined via X-Ray Fluorescence (XRF) analysis of PM samples collected immediately upstream of the Filtered Air (FA) and Traffic-Related Air Pollution (TRAP) exposure chambers at the Facility for Roadway Air Pollution Exposure (FRAPE); error bars represent ± standard error of the mean. *Lines/markers/right axis*: study-averaged percent of total PM_2.5_ mass concentration accounted for by each of the (**a**) road dust crustal elements and (**b**) traffic-related metals for the FA (gray line) and TRAP (black line) exposure chambers; error bars represent ± propagation of uncertainties in standard error of the mean. Filter-based PM samplers (8) owned and maintained by the Interagency Monitoring of Protected Visual Environments (IMPROVE) were deployed in this study to collect 24-hour continuous PM samples every third day for the study duration. Gravimetric analysis of the collected filter samples was performed according to the standard operating procedures of the IMPROVE Particle Monitoring Network (9) while a subset of these filters were chosen for PM elemental mass concentration measurements via XRF according to the measurement and QA/QC protocols of the IMPROVE Particle Monitoring Network (12). On a particle mass basis, road dust crustal elements result almost exclusively from soil-derived and clay forming minerals while traffic-related metals result largely from tire wear, brake wear and asphalt abrasion.

**References for supplemental methods**

1. Miraux S, Massot P, Ribot EJ, Franconi JM, Thiaudiere E. 3D TrueFISP imaging of mouse brain at 4.7T and 9.4T. J Magn Reson Imaging. 2008;28(2):497-503

2. Scheffler K, Lehnhardt S. Principles and applications of balanced SSFP techniques. Eur Radiol. 2003;13(11):2409-18

3. Bangerter NK, et al. Analysis of multiple-acquisition SSFP. Magn Reson Med. 2004;51(5):1038-47

4. Kruger L, Saporta S, Swanson LW. Photographic atlas of the rat brain: the cell and fiber architecture illustrated in three planes with stereotaxic coordinates. Cambridge: Cambridge University Press; 1995.

5. Oishi S, et al. Usp9x-deficiency disrupts the morphological development of the postnatal hippocampal dentate gyrus. Sci Rep. 2016;6:25783.PMC4867638

6. Keil KP, Sethi S, Wilson MD, Chen H, Lein PJ. In vivo and in vitro sex differences in the dendritic morphology of developing murine hippocampal and cortical neurons. Sci Rep. 2017;7(1):8486.PMC5559594

7. Livak KJ, Schmittgen TD. Analysis of relative gene expression data using real-time quantitative PCR and the 2(-Delta Delta C(T)) Method. Methods. 2001;25(4):402-8

8. Davis U. UCD IMPROVE Standard Operating Procedure 152 Version 2.2. Installation of Samplers 2017 [Available from: <http://vista.cira.colostate.edu/Improve/particulate-monitoring-network/>].

9. Davis U. UCD IMPROVE Standard Operating Procedure 251 Version 3.2. Sample Handling 2017 [Available from: <http://vista.cira.colostate.edu/Improve/particulate-monitoring-network/>].

10. Institute DR. DRI Model 2015 Multiwavelength Carbon Analysis (TOR/TOT) of Aerosol Filter Samples – Method IMPROVE A; 2-226r1 v1 2016 [Available from: <http://vista.cira.colostate.edu/Improve/carbon-analysis/>].

11. Turpin BJ, Lim HJ. Species contributions to PM2.5 mass concentrations: Revisiting common assumptions for estimating organic mass. Aerosol Science and Technology. 2001;35(1):602-10

12. Davis U. UCD IMPROVE Standard Operating Procedure 301 Version 2.2. X-Ray Fluorescence Analysis of Aerosol Deposits on PTFE Filters (with PANalytical Epsilon 5)", 2017 [Available from: <http://vista.cira.colostate.edu/improve/wp-content/uploads/2019/06/IMPROVE-SOP-301_Technical-Instructions-A-E_06.2019.pdf>].
